# Large field-of-view non-invasive imaging through scattering layers using fluctuating random illumination

**DOI:** 10.1038/s41467-022-29166-y

**Published:** 2022-03-18

**Authors:** Lei Zhu, Fernando Soldevila, Claudio Moretti, Alexandra d’Arco, Antoine Boniface, Xiaopeng Shao, Hilton B. de Aguiar, Sylvain Gigan

**Affiliations:** 1 Laboratoire Kastler Brossel, ENS–Université PSL, CNRS, Sorbonne Université, College de France, 24 Rue Lhomond, F-75005 Paris, France; 2 School of Physics and Optoelectronic Engineering, Xidian University, Xi’an, 710071 China

**Keywords:** Microscopy, Imaging and sensing, Imaging techniques, Fluorescence imaging

## Abstract

Non-invasive optical imaging techniques are essential diagnostic tools in many fields. Although various recent methods have been proposed to utilize and control light in multiple scattering media, non-invasive optical imaging through and inside scattering layers across a large field of view remains elusive due to the physical limits set by the optical memory effect, especially without wavefront shaping techniques. Here, we demonstrate an approach that enables non-invasive fluorescence imaging behind scattering layers with field-of-views extending well beyond the optical memory effect. The method consists in demixing the speckle patterns emitted by a fluorescent object under variable unknown random illumination, using matrix factorization and a novel fingerprint-based reconstruction. Experimental validation shows the efficiency and robustness of the method with various fluorescent samples, covering a field of view up to three times the optical memory effect range. Our non-invasive imaging technique is simple, neither requires a spatial light modulator nor a guide star, and can be generalized to a wide range of incoherent contrast mechanisms and illumination schemes.

## Abstract (web summary)

The authors demonstrate non-invasive fluorescence imaging behind scattering layers beyond the optical memory effect. They achieve this by demixing speckle patterns emitted by a fluorescent object under variable unknown random illumination, using matrix factorization and a fingerprint-based reconstruction.

## Introduction

Non-invasive optical imaging has important applications in various fields ranging from biotechnology^1,2^ to optical detection^3^. However, inhomogeneous samples, such as biological tissues, scatter light, which results in a complex speckle pattern on the detector^4,5^. With increasing depth, separating the low amount of ballistic light from the scattered light becomes a big challenge^6,7^. Over the years, many approaches have been put forward to overcome this problem by exploiting or suppressing the scattered light. With the development of spatial light modulators (SLMs), multiple ways to control and manipulate scattered light have been developed^8,9^. Several techniques have been proposed to focus light by making use of feedback signals to optimize the incident wavefront to recreate a focus that is then used for raster-scanning microscopy^10,11^. These techniques require access to both sides of the scattering layer to optimize the wavefront, which strongly limits their application in real-case scenarios. To overcome this, other strategies have been proposed based on wavefront shaping and various feedback signals such as fluorescence or ultrasound signals^11–15^. However, these approaches either require long acquisition times, entail the use of interferometric detection systems, or are limited to small fields of view (FoV). On the other hand, several techniques exploiting the angular speckle correlations, known as the optical memory effect (ME)^16–18^, have also been proposed for imaging objects hidden behind scattering media^19,20^. While these approaches are fast, their FoV is still limited by the ME range.

Linear fluorescence is widely used in biology and biomedical sciences^21–23^. It enables imaging of cellular, subcellular, or molecular components, and has the advantages of high spatial resolution, contrast, and speed. Recent advances have allowed both focusing and imaging through scattering media using fluorescent light. Even so, these methods either rely on the use of guide stars^11^, are limited to the ME range^24^, or need to characterize the scattering medium^25^.

Here, we present a robust approach that allows to non-invasively image through static scattering layers far beyond the ME range. In comparison to previous works, the only requisite for our method to work is to generate changeable random illumination patterns at the sample plane. Given that this neither requires to characterize the medium transmission matrix nor focus light through it, its implementation can be achieved without the use of an adaptive optics or wavefront shaping system (for example, by using a rotating diffuser). Also, the image retrieval process is based on a deconvolution technique instead of previously used phase retrieval approaches, which simplifies the whole method. Once excited, each fluorescent emitter generates a unique speckle pattern on the detector, which constitutes its fingerprint. Each image captured by the camera is an incoherent sum of the fingerprints from the emitters, with different relative weights due to the variable random illumination patterns and sample structure. To retrieve each individual fingerprint, we capture a set of images while randomly changing the illumination, and use a Non-negative Matrix Factorization (NMF) algorithm to demix the set of acquired frames. After that, the fingerprints are used to reconstruct the final image by exploring the correlations between them. To validate the technique, we experimentally demonstrate our non-invasive approach both on fluorescent beads and on continuous fluorescent objects.

## Results

**Fig. 1:**
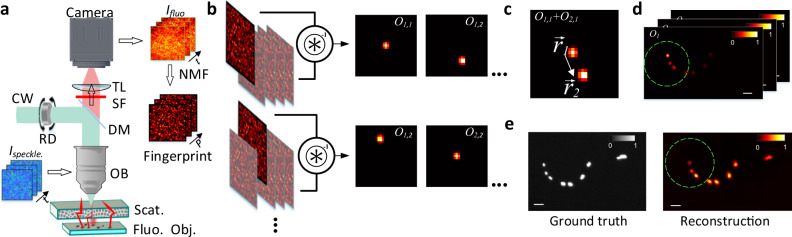
Schematic of the experimental setup and reconstruction principle. **a** Schematic view of experimental setup. A coherent light source illuminates a rotating diffuser in order to excite the fluorescent object through a scattering medium with a random modulated speckle pattern. Once excited, the emitted signal from the fluorescent objects is recorded with a camera. *I*_*fluo*_ is a series of *t* fluorescent speckles corresponding to different random speckle illuminations. The fingerprints can be recovered from *I*_*fluo*_ by using NMF. Fingerprint-based reconstruction. **b** Pairwise deconvolution (labeled as ⊛^−1^) between all the possible pairs of emitter fingerprints is performed. **c** The result of each deconvolution provides the relative position between one emitter and its neighbors. **d** By adding the resulting images for each emitter, it is possible to recover a partial image of the object centered at that emitter (see Eq. (4)). **e** All the partial images can be merged into the final reconstruction according to the relative position between neighboring emitters. Dashed circle indicates the optical memory range. Scale bar sizes are 10 μm. RD: rotating diffuser, DM: dichroic mirror, OB: objective, Scat.: scattering medium, Fluo. Obj.: fluorescent object, SF: spectral filter, TL: tube lens.

The experimental setup is depicted in Fig. 1a. A rotating holographic diffuser modulates the incident light coming from a laser by adding a random phase when the light propagates through it. Then, the modulated light travels through the scattering medium and generates a random unknown speckle pattern which illuminates the object. Each speckle grain of this pattern induces a fluorescent response from the object. This fluorescent signal propagates back through the medium, generating a unique speckle pattern on the detector (which we refer to as “fingerprint"). Each fingerprint being generated by a different fluorescent object, they are incoherent with each other. Given that multiple speckle grains illuminate different spatial regions of the object at the same time, a different incoherent sum of all fingerprints is measured by the camera for each orientation of the rotating diffuser. Although the captured images are low-contrast, random, and seemingly information-less, they contain all the fingerprints from the independent emitters of the object, but with time-varying weights. Furthermore, independent emitters within the ME range will produce correlated but shifted fingerprints on the camera^16^, while emitters outside the ME range will produce totally uncorrelated fingerprints. For a given speckle illumination, the captured image, *I*_fluo_, can be expressed as a linear superposition of those fingerprints with different weights. Thus, the camera image is given by:

1 $${I}_{fluo}(r,t)=\mathop{\sum }\limits_{k=1}^{P}{w}_{k}(r){h}_{k}(t),$$

where *I*_*fluo*_(*r*,*t*) corresponds to a low contrast speckle for the *t*th illumination, *r* is the spatial coordinate, *w*_*k*_(*r*) represents the fingerprint of the *k*th independent emitter of the object, *h*_*k*_(*t*) stands for the amount of excitation light at the *k*th emitter during the *t*th illumination, and *P* is the number of independent emitters. Given enough different random illuminations, a collection of frames can be used to retrieve each individual fingerprint, *w*_*k*_(*r*), by using an NMF algorithm, that we will now explain in detail.

### Fingerprint demixing procedure

After randomly exciting the object with a variety of *t* random speckles, a series of camera images, *I*_*fluo*_(*r*, *t*), are collected. It is possible to retrieve the fingerprints, *w*_*k*_, corresponding to each independent emitter, from the measurements *I*_*fluo*_(*r*, *t*) by finding the solution to the minimization problem^26^:

2 $$\mathop{\min }\limits_{W > 0,H > 0}{\left\Vert I-WH\right\Vert }_{F}^{2},$$

where $| | M| {| }_{F}=\sqrt{{\sum }_{i}{\sum }_{j}| {M}_{ij}{| }^{2}}$ stands for the Frobenius matrix norm. This minimization problem can be formulated as a low rank factorization, where the matrix $I\in {{\mathbb{R}}}_{+}^{r\times t}$ contains all the *I*_*fluo*_(*r*, *t*), can be approximated with two real positive matrices $W\in {{\mathbb{R}}}_{+}^{r\times \rho }$ (the fingerprints) and $H\in {{\mathbb{R}}}_{+}^{\rho \times t}$ (the temporal evolutions), where *r* are the pixels, *ρ* is the estimated rank of *I* and *t* indicates the frames. Since the collected images and the demixed fingerprints are positive, this problem corresponds exactly to the family of NMF problems. The NMF framework has been employed in demixing scenarios, both in structural imaging^25^ and functional imaging^27–29^. In our case, the estimated rank *ρ* approximately corresponds to the number of independent emitters *P* and it can be estimated from the data by minimizing the root mean squared residual of NMF as a function of the rank (see Supplementary 1).

### Fingerprint-based reconstruction

After the demixing step, the fingerprints are retrieved. Due to the ME, emitters close to each other will produce highly correlated fingerprints, with a spatial shift that is directly determined by their relative position^20^. By exploring these correlations, a position map of the emitters can be recovered, thus yielding an image of the object. Several approaches can be used to explore the correlations and calculate the shifts between fingerprints. Usually, this process is performed by doing a cross-correlation between the fingerprints and locating the position of the maximum^25^. However, here we introduce a novel approach based on deconvolution, that we denote as Fingerprint-based Reconstruction (FBR). Compared to the cross-correlation procedure, we found that this approach allows to suppress noise and strongly improves the quality of the reconstruction.

No matter if two emitters are within the same ME patch or not, one can perform the pairwise deconvolution of the *i-th* emitter by the *k-th* emitter, which can be written as:

3 $$\mathop{{{{{{\mathrm{arg}}}}}}\,{{{{{\mathrm{min}}}}}}}\limits_{{o}_{i,k}}\frac{\mu }{2}{\left|\left|{w}_{i}-{o}_{i,k} \,\circledast\, {w}_{k}\right|\right|}_{2}^{2}+{\left|\left|{o}_{i,k}\right|\right|}_{TV},$$

where *μ* is a regularization parameter, ⊛ denotes the convolution operator, $| | {{{{{{{\bf{f}}}}}}}}| {| }_{2}=\sqrt{{\sum }_{i}| {f}_{i}{| }^{2}}$ indicates the *L*_2_ vector norm, and $| | {{{{{{{\bf{f}}}}}}}}| {| }_{TV}={\sum }_{i}\sqrt{{[{D}_{x}{{{{{{{\bf{f}}}}}}}}]}_{i}^{2}+{[{D}_{y}{{{{{{{\bf{f}}}}}}}}]}_{i}^{2}}$ represents the Total Variation norm (*D*_*x*_ and *D*_*y*_ are the forward finite-difference operators along the horizontal and vertical directions). The two fingerprints are denoted as *w*_*i*_ (considered as the “image") and *w*_*k*_ (considered as the “point spread function", or PSF). When the two emitters lay within one ME range, the pairwise deconvolution yields a uniform image with a narrow delta-like peak, which is located at a distance from the center given by the relative position of the two emitters (**r**_*i*,*k*_ = **r**_*i*_ − **r**_*k*_). If the two emitters are located beyond the ME range, the deconvolution yields noise.

For a given emitter *k*, it is possible to obtain, *O*_*k*_, the partial image of the object in the vicinity of the emitter, by simply adding the result of all the pairwise deconvolutions related to that emitter, *o*_*i*,*k*_. (see Fig. 1b–d).

4 $${O}_{k}=\mathop{\sum }\limits_{i=1}^{\rho }{o}_{i,k},$$

Even if the ensemble of emitters expands well beyond the ME range, the full spatial distribution can be recovered if the different isoplanatic patches are “connected" by emitters (see Fig. 1d). For example, if emitters *i* and *k* are beyond the ME range but emitter *j* is between them, we can always calculate the shift between them as **r**_*i*,*k*_ = **r**_*i*,*j*_ + **r**_*j*,*k*_. The global reconstruction *O*^*Global*^ can be obtained by composing all the partial images, *O*_*k*_, into one image, taking into account their relative positions with respect to the first emitter, ${\overrightarrow{r}}_{k,1}$:

5 $${O}^{Global}=\mathop{\sum }\limits_{k=1}^{\rho }{O}_{k}({{{{{{{\bf{r}}}}}}}}-{{{{{{{{\bf{r}}}}}}}}}_{k,1}),$$

**Fig. 2:**
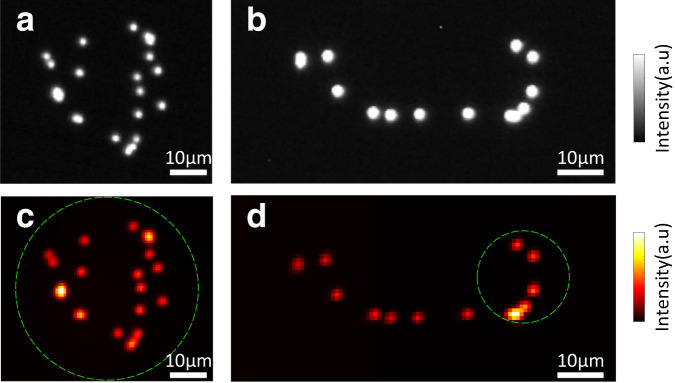
Experimental results of imaging through a scattering medium with fluorescent beads. **a**, **b** Fluorescent images of beads recorded without scattering medium. **c**, **d** Reconstruction of the object using NMF + FBR approach. The estimated rank of NMF is *ρ* = 26 for (**c**) and *ρ* = 16 for (**d**). In both cases, *t* = 5120 fluorescent speckle patterns are captured. The exposure time of **c**, **d** is set to 15 ms and 20 ms, respectively. Dashed circles indicate the optical memory effect range.

**Fig. 3:**
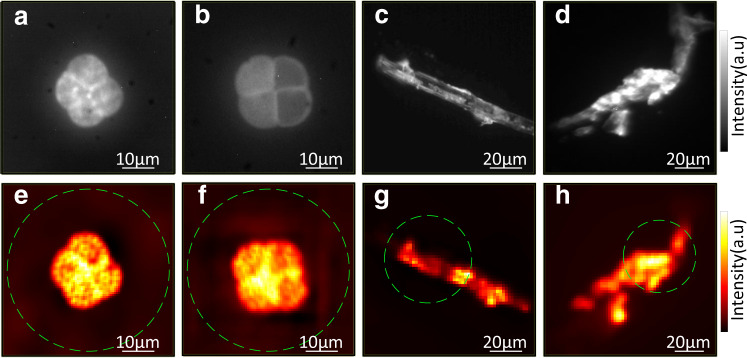
Experimental results of imaging through scattering media with continuous objects. Fluorescent images of different pollen seed structures (**a**, **b**) and different cellulose fiber structures (**c**, **d**) recorded without scattering medium. **e**–**h** Reconstruction of the objects with the NMF + FBR approach. The estimated rank for the NMF is *ρ* = 68 for (**e**), *ρ* = 85 for (**f**), *ρ* = 45 for (**g**), and *ρ* = 55 for (**h**), respectively. In both cases, *t* = 5120 fluorescent speckle patterns are recorded with an exposure time of 10 ms. Dashed circle indicates the optical memory effect range.

Experimentally, we prove that our technique can be used to recover very sparse objects by using 2D distributions of beads with a diameter of 1 μm. As shown in Fig. 2, our approach can reconstruct objects that span about three times the ME range without constraints. Non-sparse, continuous objects are common in scattering biological samples, and this often poses a difficult challenge for non-invasive imaging through scattering media approaches. To demonstrate that our technique can also work with non-sparse and continuous objects, we use fluorescence-stained pollen grains and cellulose fibers, whose reconstructed images are shown in Fig. 3. The size of fluorescence-stained pollen grains in Fig. 3a, b is smaller than the ME range and the process of reconstruction used for them is the same as what is presented in Fig. 1. For the cellulose fibers, we acquired either a single Fig. 3c or a small bundle Fig. 3d of fibers, with sizes of about two times the ME range.

In both Figs. 2 and 3 we report the reconstruction of hidden beads distributions and continuous extended objects. However, the more complex the objects are, the larger the required number of independent illuminations. Indeed, given more speckle patterns, NMF is able to provide more reliable fingerprints, thus a more fidel reconstruction (See Supplementary Fig. 7). We note that a few hundred illuminations are sufficient to recover the object reasonably well in our case. Importantly, our technique is not limited by the number of independent illuminations that we can generate with the rotating diffuser, as it is possible to produce a very large number of independent illumination with different rotating diffusers by tunning their scattering angle^29^. As an alternative to a rotating diffuser, we also propose a version of the setup using a SLM, which allows reproducible pattern projections without practical limitations on the number of patterns (see Supplementary 4).

## Discussion

Here, we report on successfully recovering the hidden objects by exploiting the correlation between the fingerprints. However, we believe, based on the spectral ME or the 3D ME, that the technique could be used to recover multi spectral or 3D objects. Another important future direction will be to explore the approach on dynamic scattering media to recover hidden objects inside of it. In that regard, our technique, in contrast with the ones based on the optical transmission matrix, does not require to characterize the medium to retrieve an image, as it only relies upon the varying video frames generated by the random illumination. However, while our approach could use the dynamic medium itself to generate random varying illumination patterns onto the embedded object, the NMF algorithm assumes that the fingerprints do not change during the acquisition process. To solve this, novel unmixing strategies, taking into account the dynamics of the system, should be explored.

In conclusion, we have shown a non-invasive technique to computationally retrieve images of objects hidden behind a static scattering medium from low-contrast fluorescent speckles using random illumination. We have demonstrated that our approach works with both sparse and continuous objects, even beyond the ME range, over previous autocorrelation approaches. Importantly, the proposed approach neither relies upon ballistic light nor uses wavefront shaping, and it is adaptable to various scattering media and objects. Our technique is flexible, robust, and opens a promising avenue towards deep fluorescence imaging in highly scattering media. Finally, it can be generalized to a wide range of incoherent contrast mechanisms and illumination schemes.

## Methods

### Experimental setup

A continuous-wave laser (*λ* = 532 nm, Coherent Sapphire) is expanded and illuminates the rotating holographic diffuser (Edmund, DG10). Then the modulated light is delivered onto the fluorescent sample through a 200 mm lens (LA1708-A, Thorlabs) and objective (Zeiss W “Plan-Apochromat" × 20, NA 1.0). After excitation, the fluorescence is scattered by the medium and collected with a 150 mm tube lens (L, AC254-150-A, Thorlabs), which is employed to produce an image onto the detector, a sCMOS camera (Hamamatsu ORCA Flash). Two dichroic filters (short pass 532 nm, Thorlabs and 533 nm notch MF525-39, Thorlabs) are used to block any signal that does not come from the fluorescence emission. The fluorescent objects, which are made of orange beads (540/560 nm, Invitrogen FluoSpheres, size 1.0 μm) or pollen seeds (Carolina, Mixed Pollen Grains Slide, *w*.*m*.), are placed below the scattering medium. The distance between the scattering medium and the fluorescent objects is 0.2 mm. A transmission pathway that consists of a microscope objective (Olympus ”MPlan N” × 20, NA 0.4), a 150 mm tube lens (L, AC254−150−A, Thorlabs), and CCD camera (Allied Vision, Manta), is used as a passive control only. This control part is used to correctly select the position of fluorescent object with a white light source (Moritex, MHAB 150W) and it also allows us to align the experimental setup. For the scattering medium, we either use a single holographic diffuser (Newport, 10DKIT-C1,10^∘^) or a combination of two (Newport, 10DKIT-C1,10^∘^ and 10DKIT-C1,1^∘^) in order to get different memory effect ranges.

The exposure time has been set from 10 ms to 20 ms, depending on the scattering medium and the fluorescent sample. Once captured, the speckle images, which contain few tens of speckle grains, are cropped from the raw images. Then, a high-pass Fourier Gaussian filter is employed to remove the background from the cropped images and the processed data set is analyzed with the NMF algorithm to obtain the fingerprint of each emitter. The experimental setup is shown in Supplementary 5.

In Fig. 2a, b, the size of each cropped image is 70 × 72 pixels, the number of patterns *t* is 5120, the scattering medium is a holographic diffuser (Newport, 10DKIT-C1, 10^∘^), and the exposure time is 15 ms, accounting for a total measurement time of 76.8 s. In Fig. 2c, d, the size of each cropped image is 64 × 66 pixels, the number of patterns *t* is 5120, the scattering medium are two holographic diffusers (Newport, 10DKIT-C1, 10^∘^ + 1^∘^), and the exposure time is 20 ms, accounting for a total measurement time of 102.4 s. In Fig. 3, the size of each cropped image is 74 × 74 pixels for the pollen grains and 140 × 136 pixels for the cellulose fibers. The number of patterns *t* is 5120, the holographic diffuser (Newport, 10DKIT-C1, 10^∘^) is used as the scattering medium, and the exposure time is 10 ms, with a total measurement time of 51.2 s.

### NMF+fingerprint-based reconstruction algorithm

For the NMF, knowing the rank of the system is necessary. The rank *ρ* is estimated by looking at the root mean square residual ∣∣*I*_*fluo*_ − *WH*∣∣_*F*_ as a function of rank *ρ* (detailed in Supplementary 1) and minimizing it. For the NMF, a random initialization is employed. The retrieved fingerprints are used as the input data of the FBR.

## Supplementary information

Supplementary information

## Data Availability

Example datasets and the designs of the rotating diffuser can be found at https://github.com/laboGigan/speckimg^30^. Full datasets are available from the authors upon reasonable request.

## Code availability

Reconstruction codes are available at https://github.com/laboGigan/speckimg^30^.
