# Supplementary material for: Large field-of-view non-invasive imaging through scattering layers using fluctuating random illumination
Source: Nat Commun. 2022 Mar 18;13:1447. doi: 10.1038/s41467-022-29166-y (PMC8933547; doi:10.1038/s41467-022-29166-y)
Supplement: Supplementary file 1 — Supplementary information [file 41467_2022_29166_MOESM1_ESM.pdf]

Supplementary information: Large field-of-view non-invasive imaging through scattering layers using fluctuating random illumination

Lei Zhu[1,2], Fernando Soldevila[1], Claudio Moretti[1], Alexandra d'Arco[1], Antoine Boniface[1], Xiaopeng Shao[2], Hilton B. de Aguiar[1], and Sylvain Gigan[1,*]

[1]Laboratoire Kastler Brossel, ENS–Université PSL, CNRS, Sorbonne Université, College de France, 24 Rue Lhomond, F-75005 Paris, France.

[2]School of Physics and Optoelectronic Engineering, Xidian University, Xi'an 710071, China

[*]Corresponding author: sylvain.gigan@lkb.ens.fr

Supplementary I. Estimating the rank of the system

In order to operate the NMF algorithm, the rank, ρ, is a unique parameter which needs to be pre-determined. Several methods can be used to estimate this rank, such as looking at clusters using a k-means algorithm [1], or by estimating the 'elbow' of the loss function [2], [3]. In our case, we use a simple procedure based on the study of the evolution of the root mean square residual (RMSR) of the NMF for different ranks to estimate the true rank of the system. Once we acquire a dataset, it is possible to demix it by setting any desired rank ρ. For each ρ, we can estimate the quality of the NMF solution by the RMSR: $||I_{fluo} - WH||_{Fro}$. As we change the rank, the RMSR varies, reaching a minimum when the estimated rank equals the true rank of the system. Also, when overestimating the rank, it is possible to have a big variance on the result of the NMF for multiple realizations. Both these behaviors can be used to delimit the true value of the rank of the system. As an example of the procedure, we show both a simulation with different numbers of beads and the same procedure with experimental datasets in Fig.1. As can be seen in Fig.1a, the minimum value of RMSR is $\rho = 5, 10$, and 15, respectively, which is in excellent agreement with the ground truth number of sources ($5, 10$, and 15). The rank estimation procedure is performed 12 times with different random initializations in order to see the variance of the results.

Furthermore, we investigate the effect of incorrectly estimating the ranks ρ of the system on the quality of our reconstruction. We experimentally choose a fluorescent object which contains 10 beads. Then, we recover the object using our approach with different rank estimations, ranging from 6 to 15. The structural similarity index metric (SSIM) [4] is used to quantify the performance of reconstruction. As shown in Fig.2, our technique is capable of achieving reconstruction with different ranks even if the proposed rank is not fully accurate. However, underestimating the rank may lead to losing some beads in the global reconstruction. On the other hand, overestimating the rank on the NMF will produce mixed patterns that hinder the capability to obtain the relative position between different emitters. Last, it must be considered that as the number of emitters gets higher, the correct estimation becomes more difficult, but at the same time, this becomes less and less detrimental to the final reconstruction. It is also worth noticing that a slight overestimation of the rank is typically ideal. While underestimating the true rank of the system will yield to missing emitters, an overestimation will only provide additional fingerprints with no real information about the position of the emitters, which will be removed at the fingerprint-based reconstruction stage.

Supplementary II. Optical memory effect

With the purpose of proving that our technique can be applied in imaging beyond the ME, different diffusers with different scattering properties are tested. We estimate the ME range of each diffuser by exploring the correlation between the speckle patterns generated while laterally moving an emitter (a small fluorescent bead). As the source moves, the correlation between speckle patterns gradually decreases, reaching a minimum correlation when the distance is longer than the ME range. Measuring this distance allows estimating the ME range for each diffuser. As reported in Fig.3, the ME range is approximately $50\,\mu m$ and $20\,\mu m$ corresponding to diffuser #1 and diffuser #2, respectively. Our non-invasive technique not only can retrieve objects within ME range as shown in Fig.2a of the manuscript, which is possible to be recovered via autocorrelation approaches, but also can reconstruct an extended object, which could not be retrieved with autocorrelation approaches. As shown in Fig.2b of the manuscript, the size of the reconstructed extended object is around $58\,\mu m \times 22\,\mu m$ with the diffuser #2.

Supplementary III. Fingerprint-based reconstruction

As described in the main text, it is possible to reconstruct the whole object as long as the ME patches have some overlaps. To present the details of FBR, we experimentally choose a fluorescent object which contains 3 fluorescent beads and display

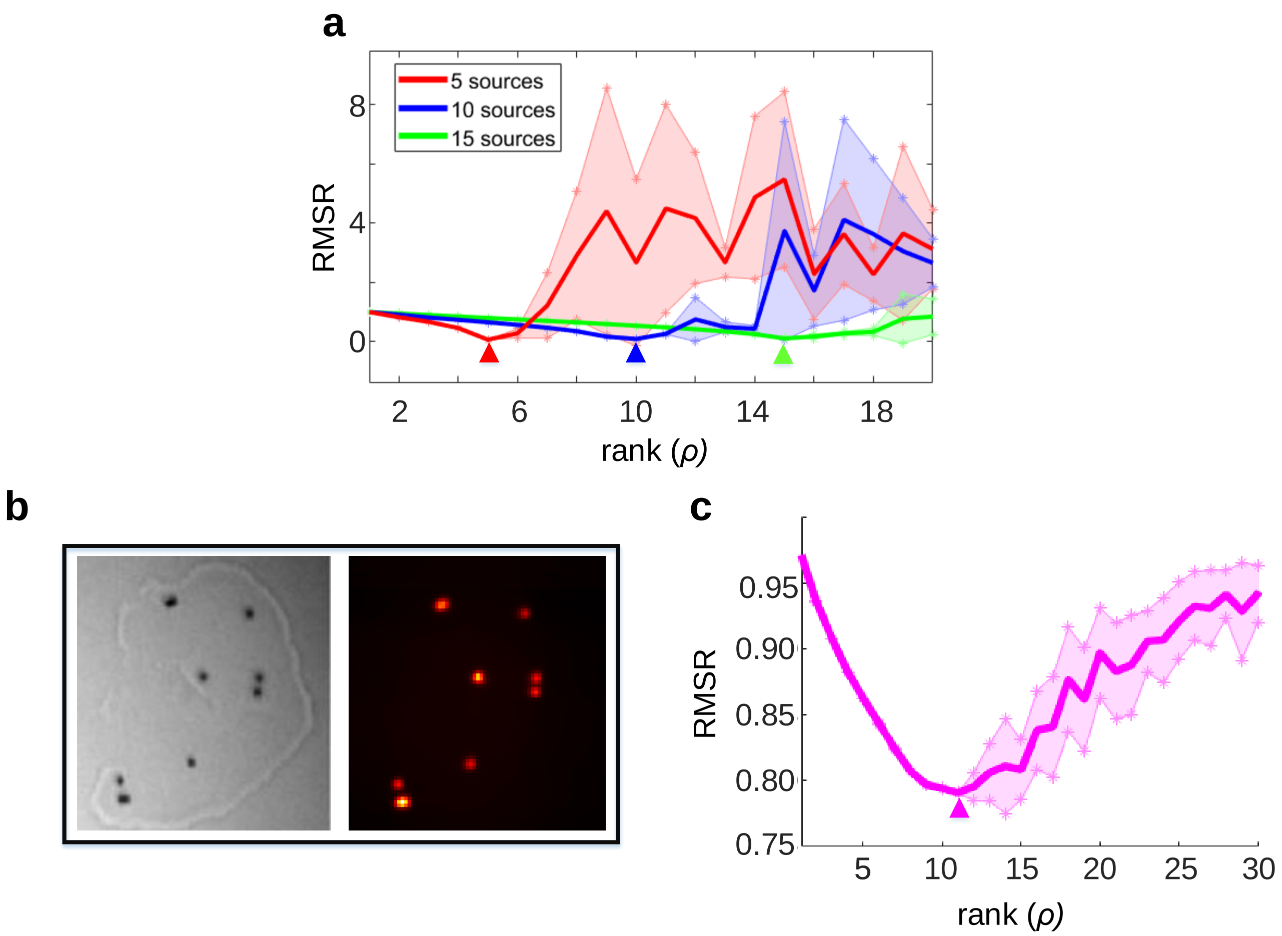

Figure 1. NMF rank estimation procedure. (a) Simulation results with P fluorescent sources. The triangle symbol marks the minimum mean value of the RMSR, the solid line stands for the mean value of the RMSR over 12 times with the random initialization value of NMF, and the asterisk is the error bar that indicates its standard deviation in both positive and negative direction. The minimum mean RMSR value is achieved when the estimated rank ρ equals the true rank P of the system. (b) Experimental results with a fluorescent object which contains $P = 11$ beads. (c) Estimating the rank of a fluorescent object shown in (b), the solid line stands for the mean value of the RMSR over 12 repetitions, the triangle symbol marks the minimum mean value of the RMSR, and the error bars indicate the standard deviation of the RMSR.

the various results from the pairwise deconvolution, $o_{i,k}$, and the different partial images, O_k. As shown in Fig.4, the O_k of the emitter k can be recovered by choosing w_k as the PSF. By looking at the maximum value of those $o_{i,k}$, the shift $\mathbf{r}_{i,k}$ between fingerprints w_i and w_k can be retrieved, as shown in Fig.1c of the manuscript. In practice, the fingerprints coming from two emitters which are beyond the ME range will not provide the relative position information of their emitters (as they will be totally uncorrelated). Thus, it is necessary to infer whether the fingerprints w_i and w_k are within or beyond the ME range. In our method, we study $\alpha = \frac{max\{o_{i,k}\}}{max\{o_{k,k}\}}$ as a function of relative distance, where $max\{o_{i,k}\}$ stands for maximum value of $o_{i,k}$. A given threshold, α_{tres} of α is introduced to evaluate it. For example, if α is greater than α_{tres}, w_i and w_k belong to the same ME range. Otherwise, they belong to different ME ranges, and their relative position cannot be retrieved. As shown in Fig.5, we experimentally investigate the maximum value of various results of pairwise deconvolutions as a function of distance between emitters. We set α_{tres} to 0.01. The full workflow of technique is depicted in Alg.1.

Supplementary IV. Producing random illumination using a SLM

Our technique retains the potential of employing a SLM for producing random illumination. The SLM can produce a large number of independent speckle illuminations. Experimentally, we replace the rotating diffuser with the SLM and perform experiments on fluorescent point-like objects and continuous volumetric objects by generating random illuminations with the SLM. The reconstruction is presented in Fig.6. To prove that it is possible to recover a more reliable image with more patterns, we show the reconstruction with the different number of patterns in Fig.7. Note that the results as shown in Fig.6 and Fig.7 are just tests to recover with the illumination patterns produced by SLM that allows generating a higher diversity of patterns than our current rotating diffuser, but it is not a limitation of our technique, as we could use different rotating diffusers to get more independent illumination patterns.

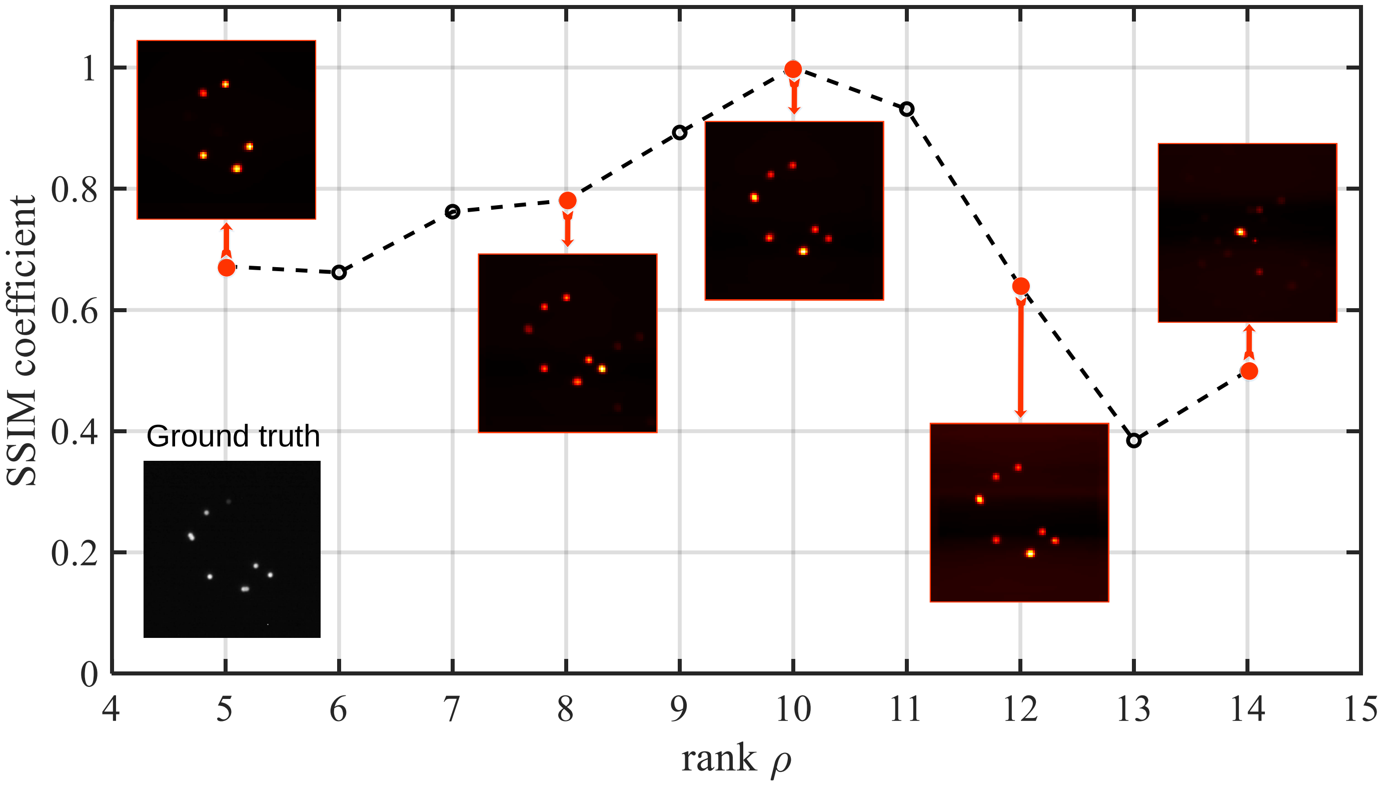

Figure 2. The plot of SSIM coefficient between different reconstructions and reference image. The reconstruction corresponding to $\rho = 10$ is treated as a reference image (the number of beads in the sample is 10).

Algorithm 1: Image retrieval procedure

Input: Series of camera images, $I_{fluo}(r, t)$.
Output: Image of the object, O^{Global}.
Estimate the rank (ρ) of the system from $I_{fluo}(r, t)$ (see Supplementary I).
Retrieve the spatial fingerprints (w_i) by using NMF with the estimated rank.
for $k = 1, ..., \rho$ **do**
  Perform the pairwise deconvolution between w_k and all the other fingerprints ($w_{i \neq k}$).
  Retrieve the relative position between emitter k and its neighbours inside the ME range.
  Calculate the partial image of the object in the vicinity of the emitter (O_k) by adding the result of all the pairwise deconvolutions related to that emitter ($o_{i,k}$).
end
Merge all the partial images (O_k) into the final reconstruction (O^{Global}) using the relative position between emitters.

Supplementary V. Deconvolution method and comparison with cross-correlation approach

Once the fingerprints have been retrieved by using the NMF algorithm, the relative position between emitters can be obtained by looking at the correlation between their fingerprints. For emitters within the ME range, the fingerprints will be highly-correlated, laterally shifted speckle patterns, while faraway emitters will present uncorrelated fingerprints. Conventionally, the lateral shift is measured by doing a cross-correlation between fingerprints. When both are correlated, a peak appears at the cross-correlation, where its distance from the center provides the shift between them. However, this method presents some drawbacks. The main issue comes from the common background envelope on the fingerprints, which even when filtered, can raise to a background in the cross-correlation, partially masking the peaks (see Fig.8.d). Moreover, when the two fingerprints are not 100% correlated, the peak tends to broaden, hindering localization accuracy. Even though it is possible to post-process this result in order to "clean" the cross-correlation (see Fig.8.e), automating the task for different scattering media, ME ranges, and signal-to-noise ratios is not a trivial process.

The rationale behind using the deconvolution approach is that a lateral shift can be expressed as the convolution of the image with a delta positioned at a distance from the center of the image equal to the shift. Then, if an image S, is a shifted version of another image, I, shifted by an amount (x_0, y_0), we have $S = I \circledast \delta(x - x_0, y - y_0)$. In that case, the shift can be retrieved by deconvolving S and I. While there are many deconvolution algorithms available, we opted for a minimization procedure

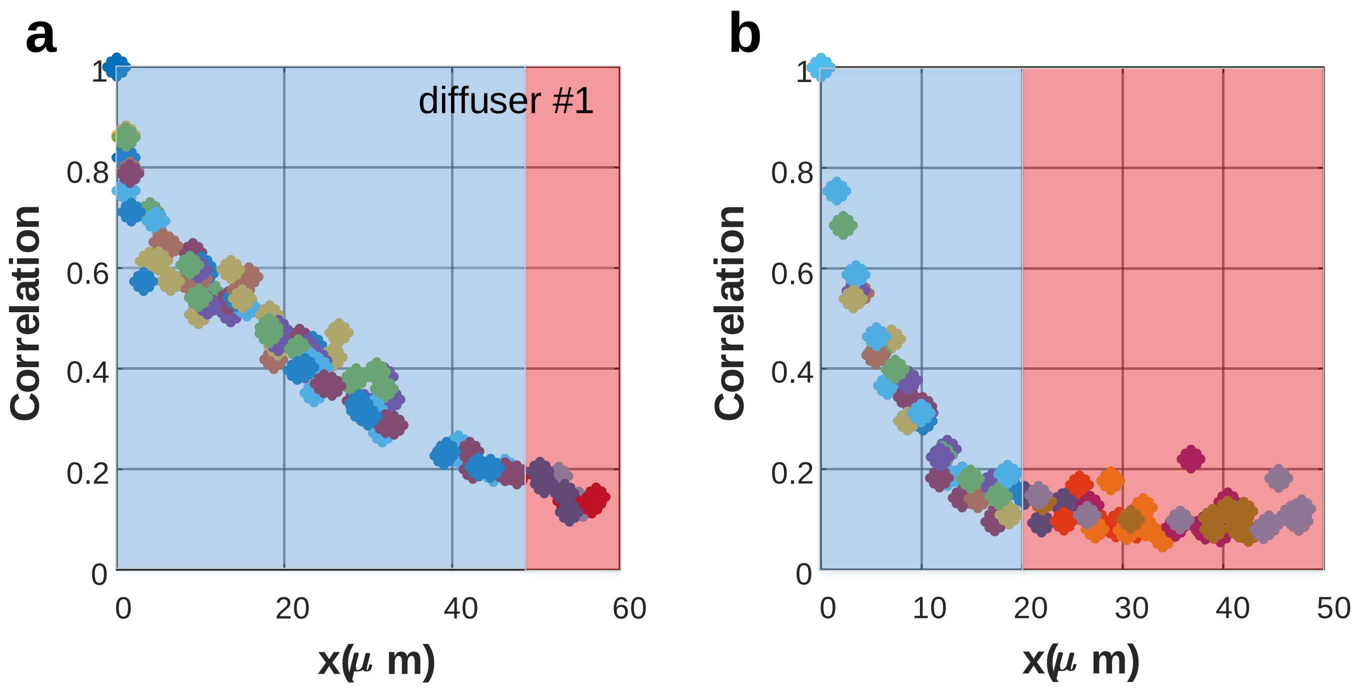

Figure 3. Plot of maximum value of cross-correlations between different patterns corresponding to different fluorescent bead positions for diffuser #1 (a) and #2 (b), where the colorcode indicates the different data sets. As a visual guide, we mark the spatial regions inside the ME range in blue, and outside in red.

adding a prior on the expected image characteristics. We chose a Total Variation (TV) minimization approach, as it promotes either delta-like or flat solutions ($\hat{x}$) with constant background, as we would expect from neighbouring or distant emitters:

$$\arg\min_{\hat{x}} \frac{\mu}{2}||S - I \circledast \hat{x}||_2^2 + ||\hat{x}||_{TV}$$

This approach evades the cross-correlation background problem (see Fig. 8.f), and allows to retrieve the final image of the object just by adding the results from all the fingerprint deconvolutions, with no additional post-processing (Fig.8.i). The minimization problem is solved by using the augmented Lagrangian method [5]. The implementation we used can be found at [6].

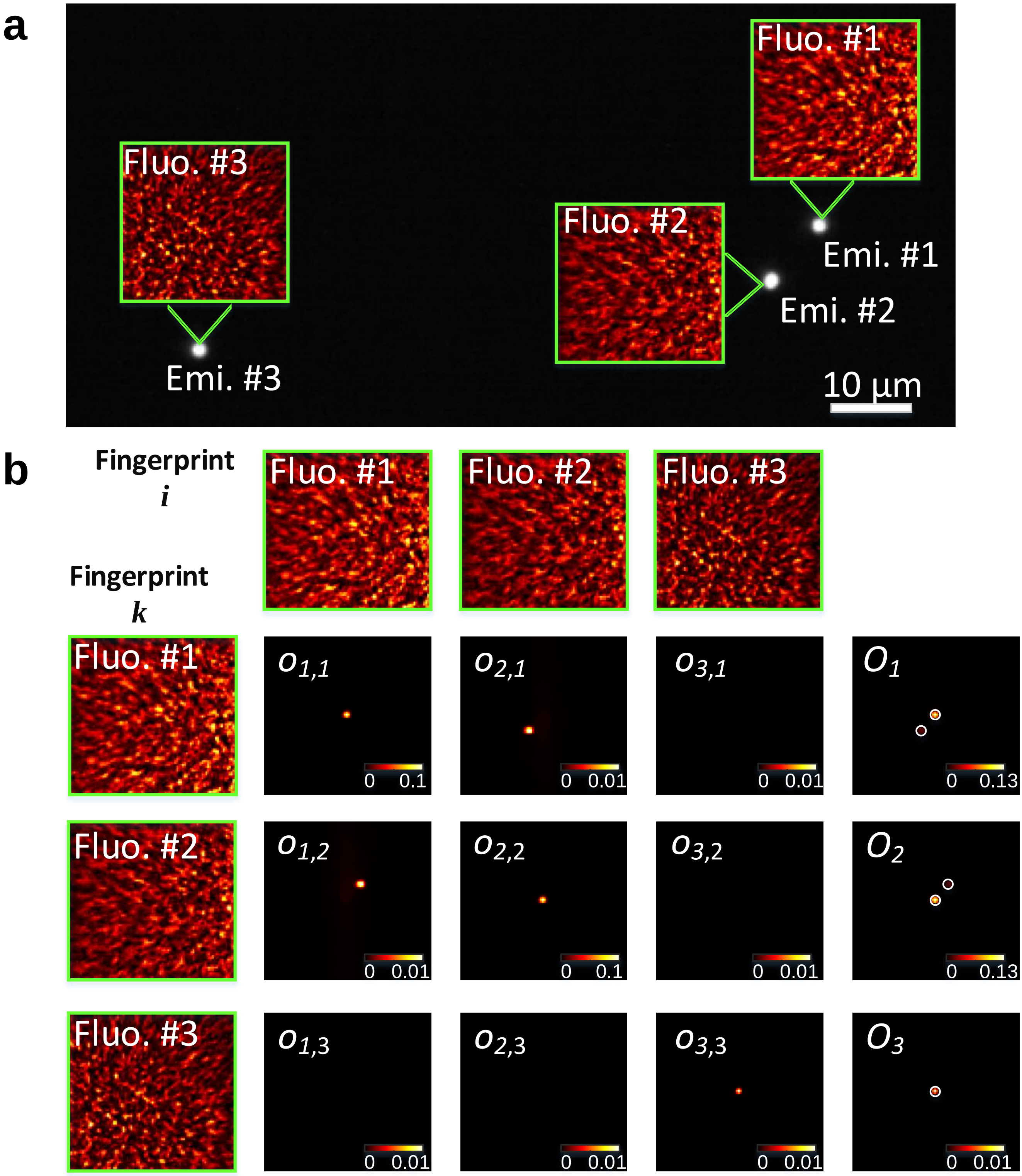

Figure 4. Detail of FBR. (a) Ground truth taken without diffuser. (b) The detail of pairwise deconvolution. The estimated rank ρ of this data set is 3 and the exposure time is $10\,\mathrm{ms}$.

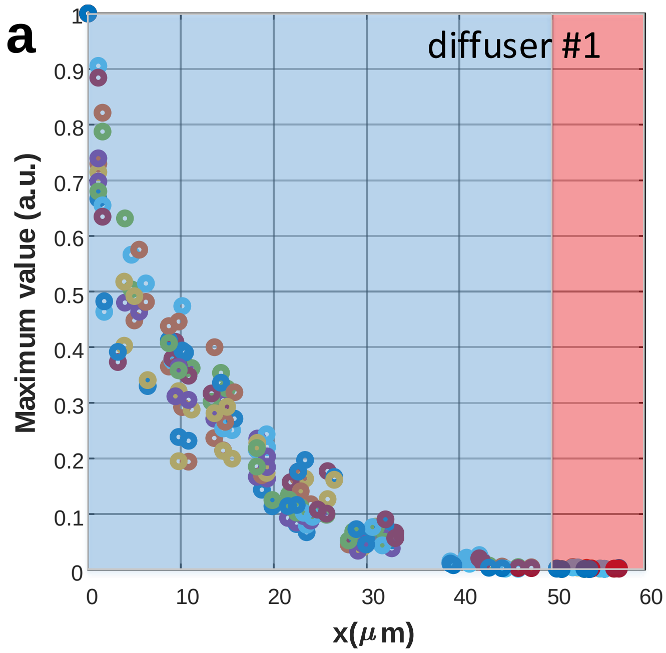

Figure 5. (a)-(b) Plot of $\alpha = \frac{max\{o_{i,k}\}}{max\{o_{k,k}\}}$ as a function of relative distance between different fluorescent emitters for diffuser #1 and diffuser #2, where the colorcode represents the different data. As a visual guide, we mark the spatial regions inside the ME range in blue, and outside in red.

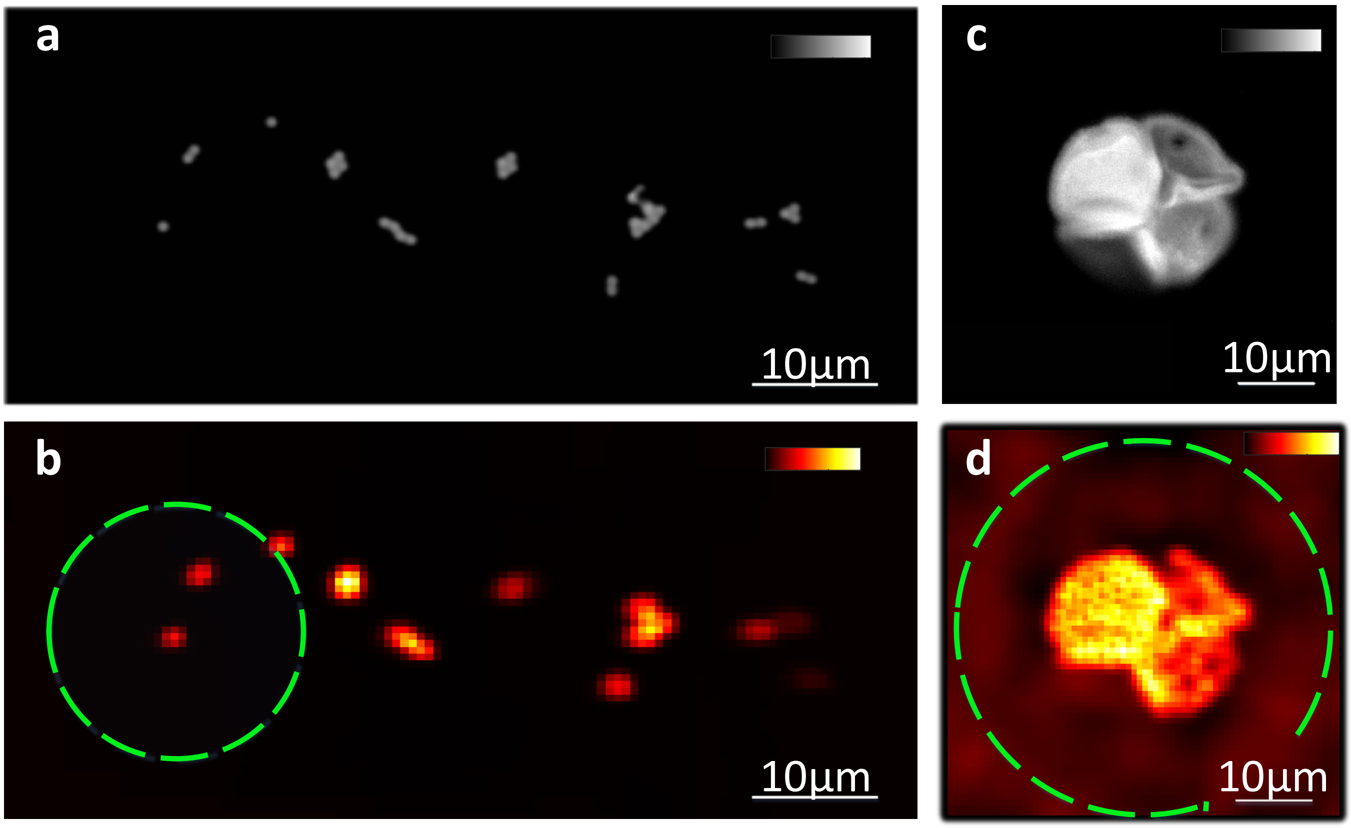

Figure 6. Experimental results of imaging through scattering media by producing random illumination with a SLM. (a,b) are ground truths of fluorescent beads object and fluorescent continuous volumetric object. (c,d) are reconstructions corresponding to (a,b), respectively. The estimated rank ρ of (c) and (d) is 14 and 53, respectively. $t = 5120$ for (c) and $t = 5120$ for (d) fluorescent speckle patterns are recorded. The dataset (c) is recorded with an exposure of 50 ms and the exposure time of (d) is 20 ms. Dashed circle indicates the optical memory effect range.

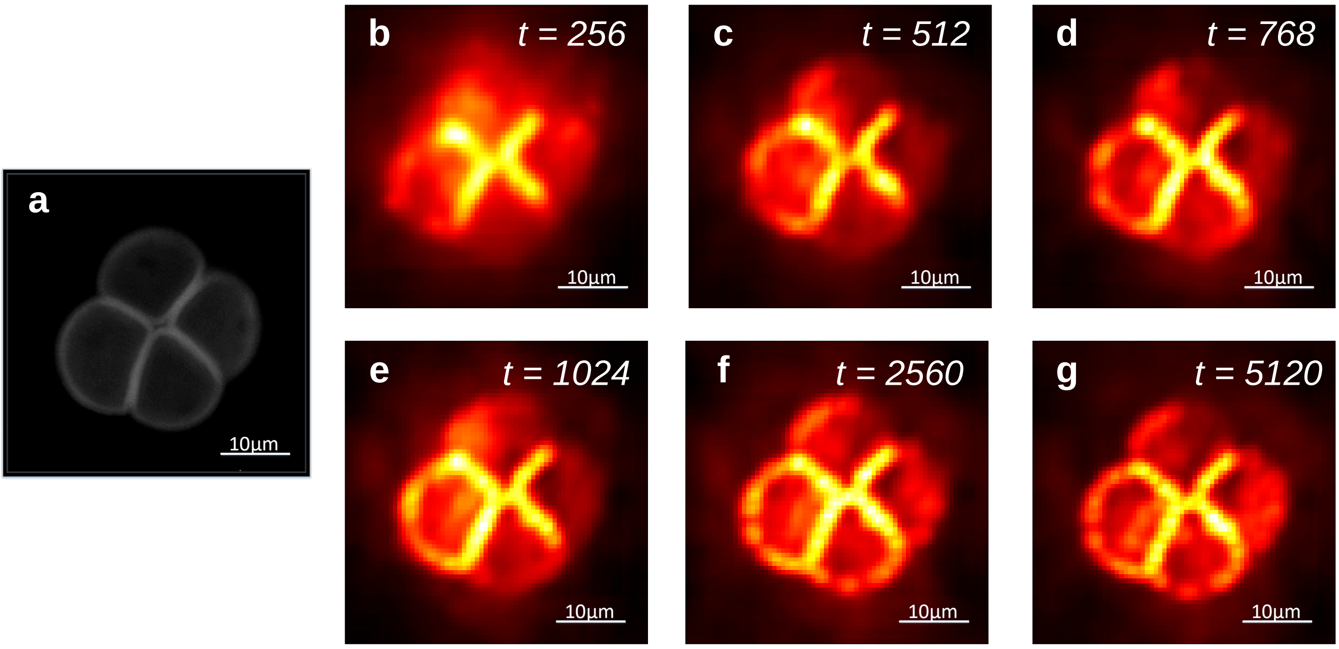

Figure 7. Performance of reconstruction with different number of speckle patterns by producing random illumination with a SLM. (a) Ground truth of fluorescent continuous volumetric object taken without scattering medium. (b)-(g) Reconstructions with different number of illumination patterns. The rank of the NMF is estimated at $\rho = 27$ for (b), at $\rho = 31$ for (c), at $\rho = 33$ for (d), at $\rho = 36$ for (e), at $\rho = 41$ for (f), and at $\rho = 53$ for (g). The dataset is recorded with an exposure of $20\,\mathrm{ms}$.

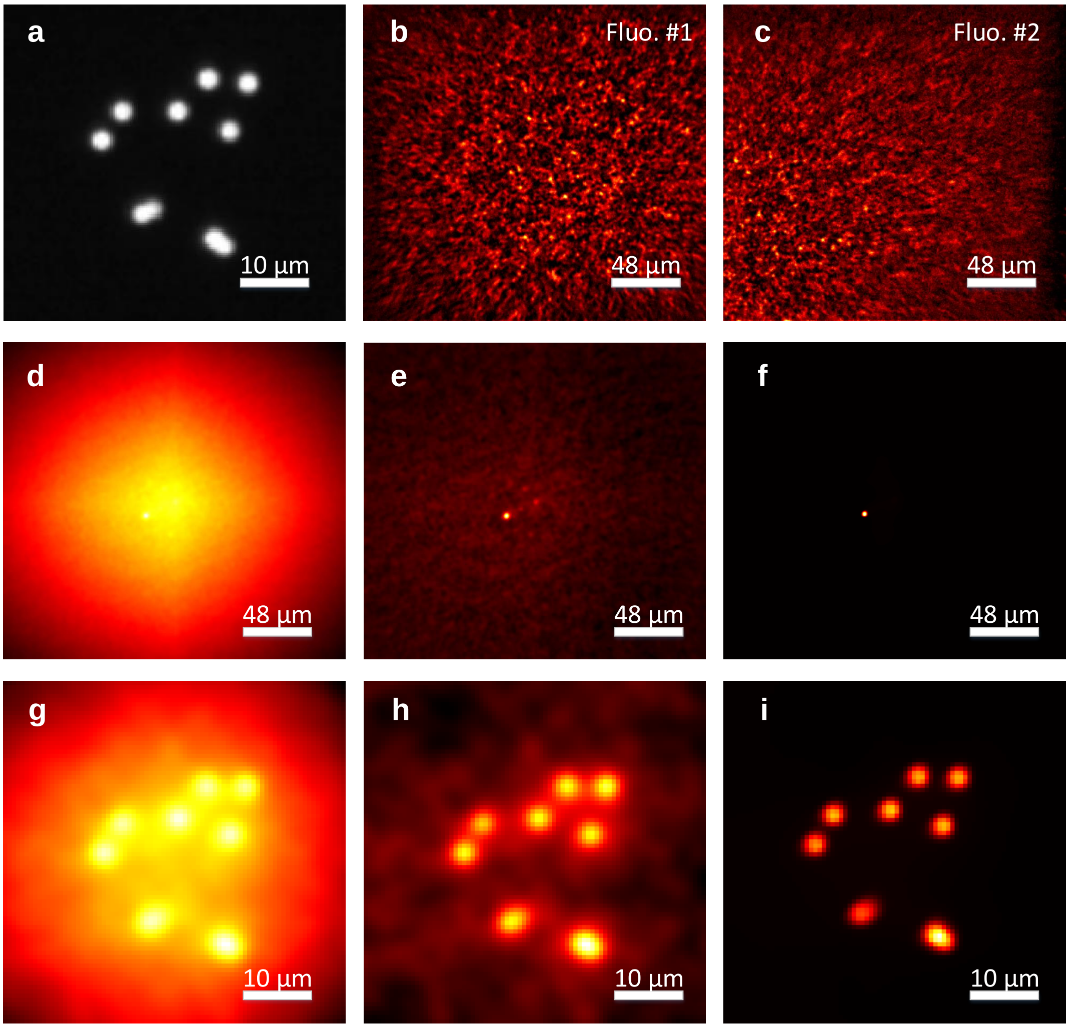

Figure 8. Deconvolution and cross-correlation retrieval comparison. (a) Ground truth object. (b,c) fingerprints for two different emitters in the object. (d) Raw cross-correlation between the two fingerprints, and its filtered version (e). Raw deconvolution between the two fingerprints. (g) Raw recovery of the object, using all the cross-correlations between the fingerprints of the object, and (h) its post-processed version (high-pass filtered). (i) Raw recovery of the object, obtained by adding the result of all the individual deconvolutions between the fingerprints, as explained in the main text.

Supplementary VI. Experimental setup

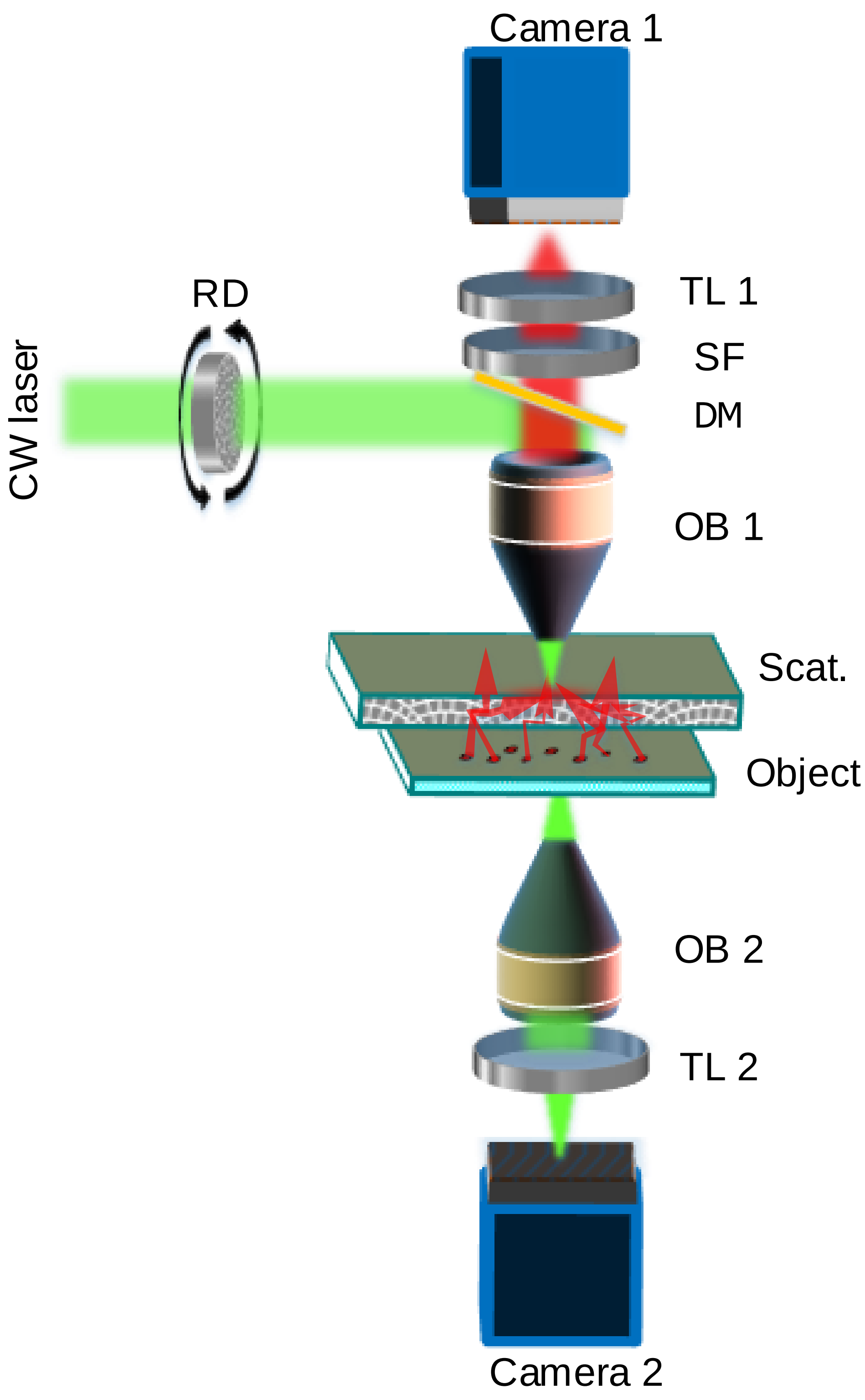

Figure 9. Experimental setup. The expanded 532 nm laser beam illuminates the rotating diffuser and the modulated light is imaged on the back focal plane of objective 1 (OB1). The object is placed in the focal plane of OB1. Camera1 is located in the imaging plane of the fluorescent imaging system that is employed to capture fluorescent speckle. The passive controlling part is made of objective 2 (OB2), tube lens 2 (TL2), and camera 2. DM: dichroic mirror, SF: spectral filter, Scat.: scattering medium.

References

[1] Moretti, C. & Gigan, S. Readout of fluorescence functional signals through highly scattering tissue. *Nature Photonics* **14**, 361–364 (2020).

[2] Boniface, A., Dong, J. & Gigan, S. Non-invasive focusing and imaging in scattering media with a fluorescence-based transmission matrix. *Nature Communications* **11**, 6154 (2020).

[3] Hutchins, L. N., Murphy, S. M., Singh, P. & Graber, J. H. Position-dependent motif characterization using non-negative matrix factorization. *Bioinformatics* **24**, 2684–2690 (2008).

[4] Wang, Z., Bovik, A., Sheikh, H. & Simoncelli, E. Image Quality Assessment: From Error Visibility to Structural Similarity. *IEEE Transactions on Image Processing* **13**, 600–612 (2004).

[5] Chan, S. H., Khoshabeh, R., Gibson, K. B., Gill, P. E. & Nguyen, T. Q. An Augmented Lagrangian Method for Total Variation Video Restoration. *IEEE Transactions on Image Processing* **20**, 3097–3111 (2011).

[6] Chan, S. H. deconvtv - fast algorithm for total variation deconvolution. https://www.mathworks.com/matlabcentral/fileexchange/43600-deconvtv-fast-algorithm-for-total-variation-deconvolution (2021). [MATLAB Central File Exchange. Retrieved October 6, 2021].
